# Cytokine upsurge among drug-resistant tuberculosis endorse the signatures of hyper inflammation and disease severity

**DOI:** 10.1038/s41598-023-27895-8

**Published:** 2023-01-16

**Authors:** Pavithra Sampath, Anuradha Rajamanickam, Kannan Thiruvengadam, Alangudi Palaniappan Natarajan, Syed Hissar, Madhavan Dhanapal, Bharathiraja Thangavelu, Lavanya Jayabal, Paranchi Murugesan Ramesh, Uma Devi Ranganathan, Subash Babu, Ramalingam Bethunaickan

**Affiliations:** 1 Department of Immunology, ICMR-National Institute for Research in Tuberculosis (ICMR-NIRT), No.1. Mayor Sathyamoorthy Road, Chetpet, Chennai, 600 031 India; 2 ICMR-NIRT-NIH-International Center for Excellence in Research, Chennai, India; 3 Department of Statistics, National Institute for Research in Tuberculosis, Chennai, India; 4 Department of Clinical Research, National Institute for Research in Tuberculosis, Chennai, India; 5 Department of Clinical Pharmacology, National Institute for Research in Tuberculosis, Chennai, India; 6 Greater Corporation of Chennai, Chennai, India; 7 Government Hospital for Thoracic Medicine (GHTM), Otteri, Chennai, India

**Keywords:** Immunology, Biomarkers, Diseases

## Abstract

Tuberculosis (TB) elimination is possible with the discovery of accurate biomarkers that define the stages of infection. Drug-resistant TB impair the current treatment strategies and worsen the unfavourable outcomes. The knowledge on host immune responses between drug-sensitive and drug-resistant infection is inadequate to understand the pathophysiological differences and disease severity. The secreted proteins, cytokines display versatile behaviour upon infection with *Mycobacterium tuberculosis* (MTB) and their imbalances often tend to assist disease pathology than protection. Therefore, studying these soluble proteins across TB infection spectrum (drug-resistant TB, drug-sensitive TB, and latent TB) may unveil the disease mediated responses and unique stage specific cytokine signatures. Thus, we sought to determine the plasma cytokine levels from healthy, latently infected, drug-sensitive, and drug-resistant TB individuals. Our study revealed top 8 cytokines (IL-17, IL-1α, IL-2, IL-10, IL-5, IFN-γ, TNF-α and IL-6) and their biomarker abilities to discriminate different stages of infection.

## Introduction

The shortcoming of global tuberculosis (TB) elimination is majorly due to drug acquired resistance and latent form of infection. During 2020, the drug acquired resistance (RR/MDR/XDR) had been estimated as 18–21% while primary resistance remained 3–4%. The consistent efforts on improved testing coverage led to surge in treatment success rate (56%) associated with drop (22%) in reported cases for drug-resistant TB (DR-TB) in 2020^1^. The prevalence of DR-TB is high in India and listed one among the 30 high burden countries for DR-TB^1^. Nevertheless, there are limitations with the current diagnostic methods using contagious sputum such as time-consumption (drug sensitivity test), minimal coverage (Gene-Xpert [Rifampicin alone] and line probe assay [Rifampicin and Isoniazid]), and expensive (whole genome sequencing)^2,3^. In addition, the latently infected people are being the abundant pool for progression to active TB however protected. The heterogenous latent conditions (latency, incipient and subclinical) and the discrimination between latent infection from active TB and extrapulmonary mycobacterial infections are inconclusive with the sparsely available signatures like Interferon Gamma Release Assay (IGRA)^4^. The preventive therapy for latency is unfeasible in a large population setting. The diagnostic lag in identifying DR-TB and latent TB infection contributes to treatment delay, disease severity and jeopardy to individual’s life.

Cytokines mount protective and pathological responses with the network of pro-inflammatory, Th1 and Th2 cytokines through interaction with complex mycobacterial antigens and RD regions^5^. Previous studies determined the augmented role of pro-inflammatory cytokines as biomarker signature for disease severity, bacterial burden, culture conversion and treatment outcome^6–10^. Mycobacterial growth restriction is achieved by the interplay of cytokines and chemokines as they orchestrate the initiation, expansion, inflammation, recruitment, differentiation, activation, and localization of mononuclear cells^11–13^. Array of cytokines such as IL-12, IL-18, IFN-γ, TNF-α, and IL-6 confers early protection against both MDR-TB and DS-TB^11,14–16^. *Mycobacterium tuberculosis* (MTB) clearance in granuloma of non-human primate model rendered through the T cells with balanced secretion of pro and anti-inflammatory cytokines^17^. However, in the chronic infection setting, the balance between protection and pathology is lost due to altered expression of cytokines that accompany delayed resolution of inflammation and tissue repair^18–20^. The susceptibility to TB disease rises on mutations in cytokine receptors which ultimately lead to defective signalling and aborts the immune surveillance and bacterial killing^8^. The immunomodulatory property obliged cytokines being patented as chemotherapeutic agent for XDR-TB. Comprehensive research is needed as the clinical trials result with single cytokines are discouraging^21^.

Studies on cytokine signatures for DR-TB and latent TB is still deficient. Multiplex assays on these host immune cytokine analytes seek attention as they are sputum-less, non-invasive, blood-based, and easily accessible. Lung infiltration of those analytes and their soluble forms in circulation deliver concepts behind disease mediated pulmonary dysfunction. Therefore, we wanted to determine the infection specific plasma cytokine biomarkers across the TB spectrum (latency, drug-sensitive and drug-resistant TB). The cytokine profile significantly altered when the infection develops from latency to drug sensitive and drug resistant state. Thus, our study reveals unique plasma cytokine biomarker signatures (IL-17, IL-1α, IL-2, IL-10, IL-5, IFN-γ, TNF-α and IL-6) which is specific at different stages of TB disease.

## Methods

### Ethical approval and informed consent

The study was approved by the ICMR-National Institute for Research in Tuberculosis Ethical committee (NIRT, IEC 2015022), Chennai, India. Informed consent was obtained from all the recruited individuals. All the experiments were performed in accordance with relevant guidelines and regulations.

### Study population and design

The study comprised of healthy controls (HC) (n = 40), latently infected individuals (LTB) (n = 40), drug-sensitive TB (DS-TB) (n = 40) and drug-resistant TB (DR-TB) (n = 40). QuantiFERON-Interferon Gamma Release Assay (IGRA) was performed for the healthy individuals who are asymptomatic for TB. IGRA negative individuals are assigned as HC and IGRA positive were taken as LTB positive individuals. DS-TB and DR-TB infected participants were recruited from the corporation centres in and around Chennai based on their routine TB diagnosis. Participants diagnosed with TB by smear or culture or GeneXpert and sensitive for first line anti-TB drugs are considered as DS-TB. DR-TB participants are those who are diagnosed with TB along with resistance to rifampicin alone or isoniazid alone or combination of both (either primary or acquired resistance) that was confirmed through GeneXpert or line probe assay (LPA) or drug sensitivity test (DST). Our study, has clinically well characterized cohort which has been diagnosed for TB, exclusive of other infections and other co-morbid conditions like Diabetes Mellitus, HIV, HCV and HBV. Blood was collected at one time point from DS-TB/DR-TB groups before the treatment initiation. Plasma was separated by centrifuging blood at 2600 rpm for 10 min and stored at − 80 °C until further analysis.

### Multiplex assay

Circulating plasma levels of cytokines were measured by Luminex Magpix Multiplex Assay system (Bio-Rad, Hercules, CA) using 14 plex Luminex Human Magnetic Assay kit (R & D systems) according to the manufacturer’s protocol. The tested 14 plex panel consists of the following cytokines: IFN-γ, IL-2, TNF-α, IL-1α, IL-1β, IL-6, IL-12, IL-17, GM-CSF, IL-18, IL-4, IL-5, IL-10, and IL-13.

### Statistical analysis

Graph-Pad PRISM Version 9.0 (GraphPad Software, CA, UA) was used to analyse the statistical difference among the groups. R software version 4.2.0 (R Core Team, 2022) was used to perform random forest analysis and principal component analysis. Shapiro–Wilk normality test was performed to test the normality of the data. Cytokine concentrations are shown as median and interquartile range (IQR) and demographic characteristics as numbers and percentages. Demographic characteristics were assessed using Fishers Exact test at 5% level of significance. Statistical significance between the study groups (DR-TB, DS-TB, LTB, and HC) for hematology and cytokine observations were analysed using Dunn test corrected for multiple comparisons using Bonferroni test. The sensitivity and specificity were assessed using receiver operating characteristic curve (ROC) analysis. The importance of the cytokines was ranked through random forest (RF) analysis. The dimensionality reduction was carried out using principal component analysis (PCA) to identify the classification pattern of the ranked cytokines. Spearman correlations were carried out to understand the relationship between cytokines. Hierarchical clustering was performed to visualize the segmentation of these cytokines in the study groups using SOM module in Multi Experiment Viewer Application (http://www.tm4.org/). p < 0.05 was considered statistically significant.

## Results

### Basic characteristics

We carried out the immunological assays on plasma samples of 160 individuals. Of these 160 individuals, 40 individuals with drug-resistant Tuberculosis (DR-TB), 40 individuals with drug-sensitive tuberculosis (DS-TB), 40 individuals with latent tuberculosis (LTB) and 40 healthy control (HC) individuals. Further demographics and haematological data of participants are described in Table 1. The median age of 35 years (range 18–63) for DR-TB, 28 years (range 14–49) for DS-TB, 27 years (range 21–50) for LTB and 27.5 years (range 18–50) for HCs were not significantly different among recruited individuals. Significant differences were observed in the hematological parameters except RBC, eosinophil and basophil count were mentioned in Table 1 with their respective p values.

**Table 1:** Demographic characteristics.

	HC N = 40	LTB N = 40	DS-TB N = 40	DR-TB N = 40	p-value
Gender					
Female	20 (50)	22 (55)	17 (42.5)	10 (25)	**0.038**
Male	20 (50)	18 (45)	23 (57.5)	30 (75)	
Age in Years	27.5 (18–50)	27 (21–50)	28 (14–49)	35 (18–63)	0.225
Height (cm)	162 (153–190.5)	160.5 (151–172)	162 (130–174)	165.1 (140–176)	0.533
Weight (kg)	70 (43–84)	65 (52–86)	40 (31–64)	41 (25–79)	** < 0.001**
BMI	26.4 (16.5–31.4)	26.1 (22.9–33.8)	16.5 (12.4–24.4)	16.7 (9.7–28.3)	** < 0.001**
**Habits**					
Smoking					
No	0	0	15 (71.4)	19 (67.9)	0.788
Yes	0	0	6 (28.6)	9 (32.1)	
Unknown	40	40	19	12	
Tobacco					
No	0	0	20 (95.2)	25 (89.3)	0.451
Yes	0	0	1 (4.8)	3 (10.7)	
Unknown	40	40	19	12	
Alcohol					
No	0	0	13 (59.1)	16 (57.1)	0.890
Yes	0	0	9 (40.9)	12 (42.9)	
Unknown	40	40	18	12	
**Clinical manifestation**					
Cough					
No	0	0	2 (8.3)	0 (0)	0.119
Yes	0	0	22 (91.7)	28 (100)	
Unknown	40	40	16	12	
Expectoration					
No	0	0	5 (21.7)	3 (10.7)	0.281
Yes	0	0	18 (78.3)	25 (89.3)	
Unknown	40	40	17	12	
Hemoptysis					
No	0	0	18 (78.3)	24 (85.7)	0.487
Yes	0	0	5 (21.7)	4 (14.3)	
Unknown	40	40	17	12	
Fever					
No	0	0	13 (56.5)	10 (37)	0.168
Yes	0	0	10 (43.5)	17 (63)	
Unknown	40	40	17	13	
Difficulty in breathing					
No	0	0	11 (47.8)	13 (46.4)	0.921
Yes	0	0	12 (52.2)	15 (53.6)	
Unknown	40	40	17	12	
Chest pain					
No	0	0	19 (86.4)	27 (96.4)	0.193
Yes	0	0	3 (13.6)	1 (3.6)	
Unknown	40	40	18	12	
Loss of appetite					
No	0	0	12 (52.2)	10 (35.7)	0.238
Yes	0	0	11 (47.8)	18 (64.3)	
Unknown	40	40	17	12	
Loss of weight					
No	0	0	9 (40.9)	7 (25)	0.231
Yes	0	0	13 (59.1)	21 (75)	
Unknown	40	40	18	12	
Others					
No	0	0	20 (90.9)	28 (100)	0.103
Yes	0	0	2 (9.1)	0 (0)	
Unknown	40	40	18	12	
**Hematology parameters**					
WBC (10^3^ cells/µL)	7.8 (4.5–11.7)	7.4 (4.8–13.4)	8.1 (4.5–16.5)	9.7 (5.8–18.3)	** < 0.001**
RBC (10^3^ cells/µL)	4.6 (4–6)	4.7 (3.2–5.7)	4.4 (2.9–7.4)	4.5 (2.4–6.3)	0.180
HGB (g/dL)	13.3 (10.3–17.7)	14 (11.1–17)	11.7 (7.9–21.9)	12.3 (7.2–17.4)	** < 0.001**
HCT (%)	40.1 (31.3–51.5)	40.3 (31.8–48.2)	36 (24.8–63.7)	36.3 (9.1–51.9)	** < 0.001**
MCV (fL)	86.9 (73.1–96.5)	87.8 (76.1–100)	84.2 (52–98)	81 (37–99.1)	**0.005**
MCH (pg)	28.7 (12.1–34.4)	29.9 (25.7–36.2)	27.4 (15.9–33.9)	27 (21.2–33.2)	** < 0.001**
MCHC (g/dL)	33.6 (31.7–36.3)	34.3 (32.1–36.2)	33 (30.5–35.6)	32.9 (30.3–35.6)	** < 0.001**
RDW-CV (%)	13 (10.8–17.7)	13.3 (12–17.1)	15.5 (10.7–25.3)	16.4 (12.5–27.9)	** < 0.001**
Platelets (10^3^ cells/µL)	244.5 (110–447)	239 (144–557)	302.5 (4.1–680)	365 (83–781)	** < 0.001**
MPV (fL)	9.7 (6.8–50.7)	9.6 (8.1–12.2)	8.7 (7–11.3)	8.4 (6.7–10.8)	** < 0.001**
Neutrophil (10^3^ cells/µL)	4.5 (2–8.7)	4.2 (2.4–7.3)	5.7 (2.5–12.2)	6.6 (3.4–15.4)	** < 0.001**
Lymphocyte (10^3^ cells/µL)	2.4 (0.6–3.8)	2.6 (1.2–4.8)	1.9 (0.7–3.1)	1.7 (0.7–3.8)	** < 0.001**
Monocyte (10^3^ cells/µL)	0.4 (0.2–0.9)	0.4 (0.3–0.9)	0.6 (0.2–1.7)	0.7 (0.1–1.8)	** < 0.001**
Eosinophil (10^3^ cells/µL)	0.2 (0–2.1)	0.2 (0–1.3)	0.1 (0–1.9)	0.2 (0–2.1)	0.215
Basophil (10^3^ cells/µL)	0 (0–0.1)	0 (0–0.1)	0 (0–0.7)	0 (0–0.2)	0.266
**Ratios**					
Monocyte to lymphocyte ratio (MLR) (10^3^ cells/µL)	0.2 (0.1–1.4)	0.2 (0.1–0.3)	0.4 (0.1–1.1)	0.4 (0.2–0.9)	** < 0.001**
Neutrophil to lymphocyte ratio (NLR) (10^3^ cells/µL)	1.8 (0.9–5.4)	1.8 (0.8–5.1)	3.5 (1.4–10.2)	4.5 (1.7–10.1)	** < 0.001**
Monocyte to neutrophil ratio (MNR) (10^3^ cells/µL)	0.1 (0–0.3)	0.1 (0.1–0.2)	0.1 (0.1–0.2)	0.1 (0–0.2)	0.363

Values were presented as frequency (percentage) and median (range: minimum − maximum).

Fishers exact test and Dunn test was used at 5% level of significance.

The "Unknown" category was excluded while calculating the Fishers Exact test.

Significant values are given in bold.

### Drug-Resistant tuberculosis is associated with altered cytokine levels

**Figure 1:**
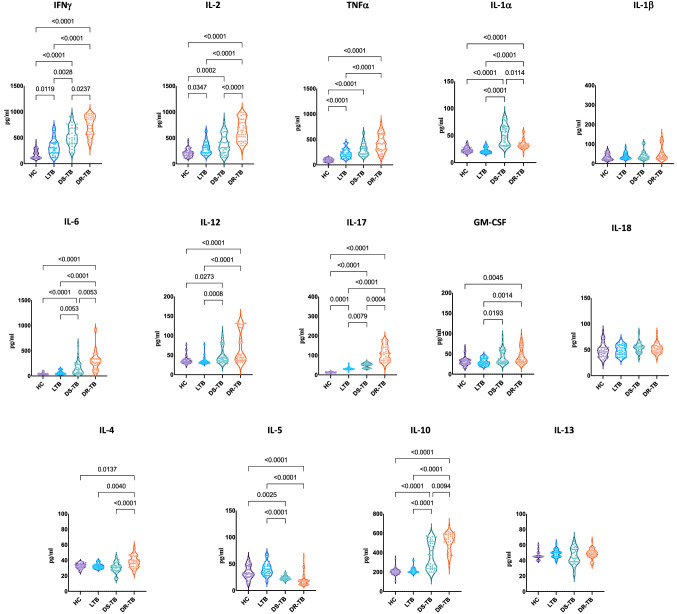
Altered cytokine profile among DS-TB and DR-TB groups compared to HC or LTB groups. Statistical differences were analysed by Dunn test corrected for multiple comparisons using Bonferroni test and significant p values < 0.05 were mentioned in the graphs.

We wanted to determine the dynamics of cytokines in different groups of infection and / or disease, therefore, they may be useful as a biomarker for diagnosis. We examined the array of cytokines using a multiplex assay profile in the plasma of all the above-mentioned groups. The cytokine concentration was shown as median and IQR and described in Table 2. As shown in Fig. 1, patients with DR-TB exhibited significantly increased levels of IFN-γ (p = 0.0237), TNF-α (p < 0.0001), IL-6 (p = 0.0053), IL-17 (p = 0.0004), IL-4 (p < 0.0001) and IL-10 (p = 0.0094) compared to DS-TB. On the contrary, IL-1α (p = 0.0114) levels were significantly decreased in DR-TB than in the DS-TB patient group.

**Table 2:** Cytokine concentration shown as median and interquartile range (Q1 and Q3) across the groups.

Cytokines	HC	LTB	DS-TB	DR-TB
IFN-g	183.2 (110.59–245.24)	305.36 (207.32–406.66)	497.01 (394.69–674.78)	771.37 (569.91–895.99)
TNF-a	207.14 (164.83–248.16)	276.63 (215.06–362.81)	318.43 (248.91–521.56)	628.71 (438.16–814.61)
IL-2	97.22 (65.32–117.08)	210.7 (166.11–279.97)	276.63 (215.06–362.81)	416.17 (296.98–545.96)
IL-6	39.73 (32.82–50.99)	54.94 (36.92–83.2)	130.11 (49.55–228.89)	277.26 (192.66–343.49)
IL-12p70	33.8 (31.02–38.66)	31.95 (30.10–35.88)	42.13 (34.73–58.13)	47.69 (37.27–120.33)
IL-17	12.27 (11.02–15.02)	30 (30–33)	52.5 (36–58.13)	108.75 (75–140.63)
GM-CSF	30.31 (25.02–34.89)	28.87 (24.06–37.79)	34.41 (28.98–55.93)	37.14 (30.34–58.06)
IL-1a	22.29 (19.45–26.34)	20.39 (18.27–27.29)	50.19 (32.09–64.99)	31.14 (28.3–36.15)
IL-4	32.91 (30.86–34.96)	31.95 (30.02–34.03)	30.98 (27.14–33.87)	36.95 (31.95–45.22)
IL-10	200 (180–220)	200 (200–225)	338.52 (236.9–507.82)	539.77 (441.23–582.71)
IL-5	31.5 (26–45)	38.25 (32–46)	23 (21–25.63)	17.51 (11.9–20)
IL-13	45 (45–48.13)	50 (45–50.63)	43.73 (38.46–54.09)	48.82 (45–52.17)
IL-18	46.08 (37.62–54.47)	47.94 (40.51–56.81)	53.51 (46.71–57.4)	51.56 (45–57.40)
IL-1beta	29.54 (21.83–50.19)	32.52 (26.1–49.56)	33.79 (27.1–48.49)	39.19 (25.55–69.81)

Further, DR-TB patients exhibited significantly increased levels of IFN-γ (p < 0.0001), IL-2 (p < 0.0001), TNF-α (p < 0.0001), IL-1α (p < 0.0001), IL-6 (p < 0.0001), IL-12p70 (p < 0.0001), IL-17 (p < 0.0001), GM-CSF (p = 0.0014), IL-4 (p = 0.0040), and IL-10 (p < 0.0001) in comparison to LTB individuals. DR-TB patients exhibited significantly increased levels of IFN-γ(p < 0.0001), IL-2 (p < 0.0001), TNF-α (p < 0.0001), IL-1α (p < 0.0001), IL-6 (p < 0.0001), IL-12p70 (p < 0.0001), IL-17 (p < 0.0001), GM-CSF (p = 0.0045), IL-4 (p = 0.0137), and IL-10 (p < 0.0001) in comparison to control group. In contrast, IL-5 (p < 0.0001) levels were significantly reduced in DR-TB compared to LTB and control groups.

DS-TB patients exhibited significantly increased levels of IFN-γ (p = 0.0028), IL-1α (p < 0.0001), IL-6 (p = 0.0053), IL-12p70 (p = 0.0008), IL-17 (p = 0.0079), GM-CSF (p = 0.0193) and IL-10 (p < 0.0001) in comparison to LTB. Also, when compared to HC group, DS-TB patients exhibited significantly increased levels of IFN-γ(p < 0.0001), IL-2 (p < 0.0001), TNF-α (p = 0.0002), IL-1α (p < 0.0001), IL-6 (p < 0.0001), IL-12p70 (p = 0.0273), IL-17 (p < 0.0001) and IL-10 (p < 0.0001). In contrast, IL-5 levels were significantly decreased in DS-TB patients in comparison to LTB (p < 0.0001) and HC (p = 0.0025) individuals.

LTB individuals exhibited significantly increased levels of IFN-γ (p = 0.0119), IL-2 (p < 0.0001), TNF-α (p = 0.0347) and IL-17 (p = 0.0001) in comparison to HC group. Thus, the clinical spectrum of TB disease/ infection is associated with altered levels of cytokines.

### Heat maps divulge tendencies in cytokine milieu in DR-TB, DS-TB, LTB, and HC

**Figure 2:**
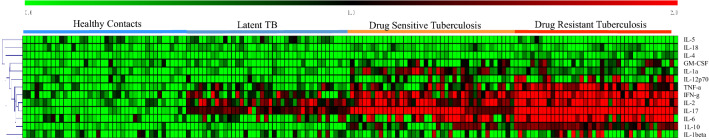
Heatmaps representing the measured cytokines and their hierarchical clustering across the TB disease spectrum by log 2 conversion and HC group mean normalization.

The trends in the cytokine expression profile were assessed by hierarchical clustering using log 2 transformed and mean normalized values. As shown in Fig. 2, heat map reveals the changes in the cytokine trend from latency to drug sensitive and to drug resistance with a greater number of cytokine accumulation during severe condition like DR-TB. Prior to infection, the disease protective latent condition presented the cytokine panel with mild or moderate levels of TNF-α, IFN-γ, IL-2, IL-17, and IL-6. In the diseased state, DS-TB individuals presented differential cytokine expression with high levels of IFN-γ, IL-2, IL-17, and IL-6; moderate levels of IL-1α, IL-12p70, TNF-α and IL-10 and mild levels of GM-CSF. DR-TB individuals are associated with abundant cytokine expression of TNF-α, IFN-γ, IL-2, IL-17, IL-6, and IL-10 and with moderate expression of GM-CSF, IL-12p70, IL-1α and IL-1β. As per the heat map, the expression of TNF-α, IL-6 and IL-10 are abundantly increased in DR-TB whereas, IL-1α expression is higher in DS-TB compared to other groups. On contrary, IL-5 expression is moderate in the LTB and HC group and low in DS-TB and DR-TB groups. Thus, these analyses help to reveal the power of cytokines to demarcate the spectrum of TB disease/infection (DR-TB, DS-TB, and LTB) from HC.

### Diagnostic performance of top 8 cytokines for bifurcation of DR-TB, DS-TB, LTB, and HC

**Figure 3:**
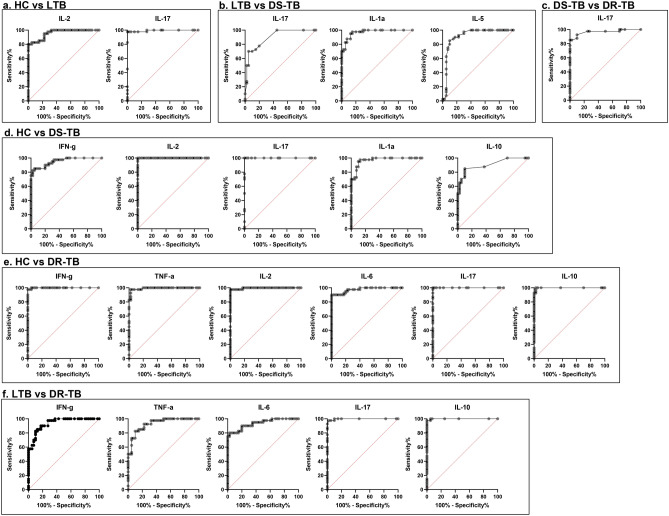
ROC curves of significant cytokines with AUC > 0.9 showing the diagnostic efficiency between the study groups, (**a**) HC vs LTB, (**b**) LTB vs DS-TB, (**c**) DS-TB vs DR-TB, (**d**) HC vs DS-TB, (**e**) HC vs DR-TB and (**f**) LTB vs DR-TB.

We conducted a ROC analysis to determine the diagnostic capabilities of each cytokine to distinguish between the groups of study. The representative curves showing the cytokines with the best diagnostic accuracy between and among these groups are shown in Fig. 3. IL-17 exhibited AUC = 0.97 and significantly (p < 0.0001) discriminate DR-TB from DS-TB (Fig. 3c). Cytokines, IFN-γ (AUC = 0.94, p < 0.0001), TNF-α (AUC = 0.93, p < 0.0001), IL-6 (AUC = 0.93, p < 0.0001), IL-17 (AUC = 1, p < 0.0001) and IL-10 (AUC = 1, p < 0.0001) differentiate DR-TB from LTB (Fig. 3f). Also, cytokines IFN-γ (AUC = 1, p < 0.0001), TNF-α (AUC = 0.99, p < 0.0001), IL-2 (AUC = 1, p < 0.0001), IL-6 (AUC = 0.98, p < 0.0001), IL-17 (AUC = 1, p < 0.0001) and IL-10 (AUC = 1, p < 0.0001) differentiate DR-TB from HC group (Fig. 3e). IL-17 (AUC = 0.90, p < 0.0001), IL-1α (AUC = 0.97, p < 0.0001) and IL-5 (AUC = 0.92, p < 0.0001) differentiate DS-TB from LTB (Fig. 3b). IFN-γ (AUC = 0.95, p < 0.0001), IL-2 (AUC = 1, p < 0.0001), IL-17 (AUC = 1, p < 0.0001), IL-1α (AUC = 0.97, p < 0.0001) and IL-10 (AUC = 0.91, p < 0.0001) differentiate DS-TB from HC (Fig. 3d). IL-2 (AUC = 0.96, p < 0.0001) and IL-17 (AUC = 0.99, p < 0.0001) differentiate LTB from HC (Fig. 3a). However, other cytokines IL-12, IL-18, GM-CSF, and IL-13 could not significantly discriminate DR-TB from DS-TB, LTB, and the control group.

**Figure 4:**
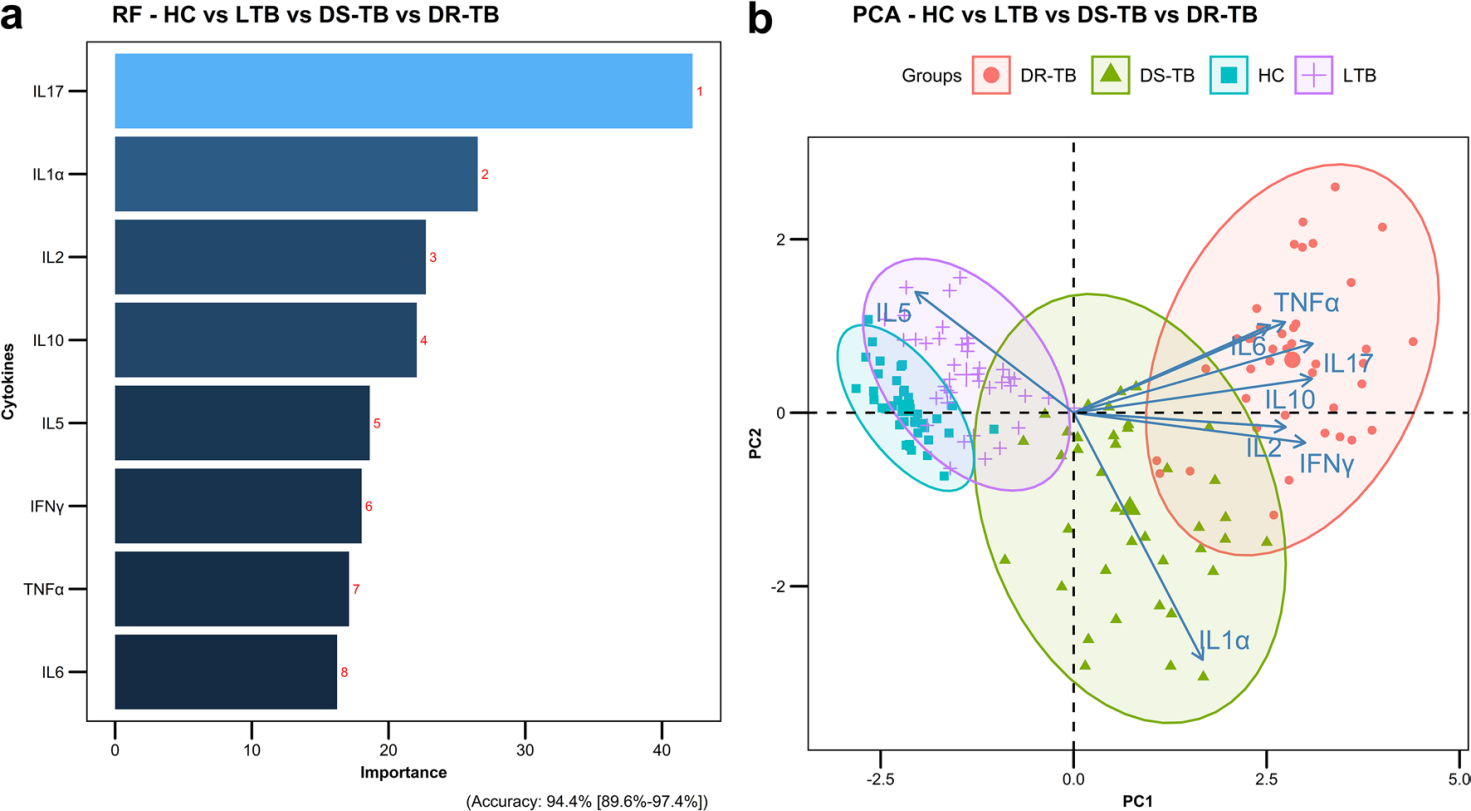
Random-forest analysis plot (**a**) and principal component analysis plot (**b**) of top 8 cytokines across the study groups (HC vs LTB vs DS-TB vs DR-TB).

**Figure 5:**
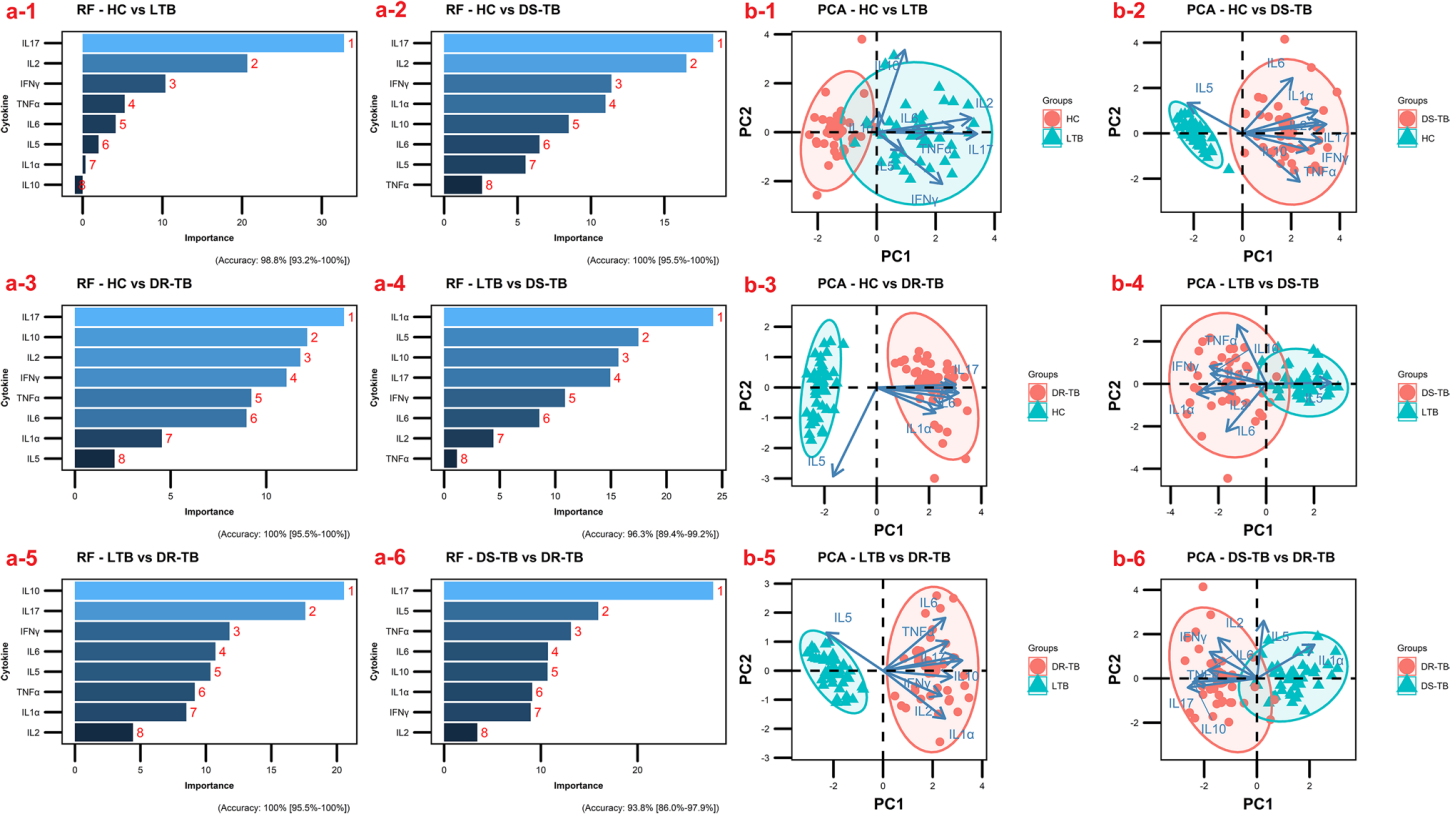
Sub-group comparisons of top 8 cytokines by Random-forest analysis (**a-1** HC vs LTB, **a-2** HC vs DS-TB, **a-3** HC vs DR-TB, **a-4** LTB vs DS-TB, **a-5** LTB vs DR-TB and **a-6** DS-TB vs DR-TB) and principal component analysis (**b-1** HC vs LTB, **b-2** HC vs DS-TB, **b-3** HC vs DR-TB, **b-4** LTB vs DS-TB, **b-5** LTB vs DR-TB and **b-6** DS-TB vs DR-TB).

**Figure 6:**
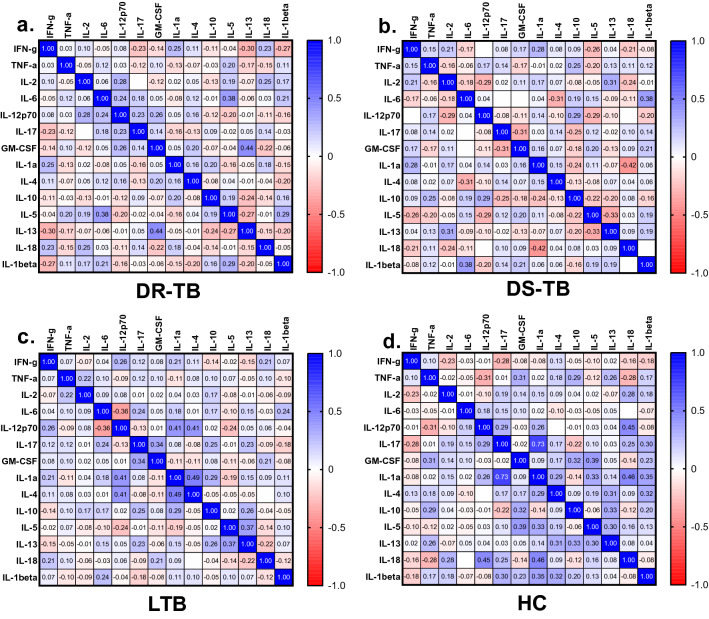
Correlation matrix using spearman rank correlation between the measured cytokines of the study groups, a. DR-TB, b. DS-TB, c. LTB and d. HC.

In addition, we performed a random forest (RF) analysis to understand the importance of these cytokines and their discrimination toward the separation of study groups. According to the order of importance, RF plots of overall comparison (HC vs LTB vs DS-TB vs DR-TB) presented IL-17, IL-1α, IL-2, IL-10, IL-5, IFN-γ, TNF-α and IL-6 as the topmost classifiers **(**Fig. 4a). This was in accordance with the ROC results where these cytokines displayed higher AUC values of above 0.8. Similarly in the subgroup comparisons, the same IL-17 was obtained as the topmost classifier for HC vs LTB/DS-TB/DR-TB (Fig. 5a-1,a-3) and DS-TB vs DR-TB (Fig. 5a-6) whereas, IL-1α for LTB vs DS-TB (Fig. 5a-4) and IL-10 for LTB vs DR-TB (Fig. 5a-5).

All cytokines were then dimensionally reduced through the principal component analysis, which resulted in a lower variation of the first two dimensions, and the ellipses of HC overlapped with LTB, DR-TB with DS-TB, and DS-TB with all three groups. To achieve better bifurcation with a minimum of 80% variance, the weaker cytokines from the RF plots were removed and the dimensionality reduction analysis were repeated. The PCA of top 8 cytokines (IL-17, IL-1α, IL-2, IL-10, IL-5, IFN-γ, TNF-α and IL-6) exhibited better separation of clusters with variances above 90%. The discriminative accuracy and the ranges are as follows: 94.4% (89.6–97.4%) for HC vs LTB vs DS-TB vs DR-TB **(**Fig. 4b) and for sub-group comparisons are 93.8% (86–97.9%) for DS-TB vs DR-TB (Fig. 5b-6), 96.3% (89.4–99.2%) for LTB vs DS-TB (Fig. 5b-4), 98.8% (93.2–100%) for HC vs LTB (Fig. 5b-1), 100% (95.5–100%) for LTB vs DR-TB (Fig. 5b-5) and HC vs DS-TB/DR-TB (Fig. 5b-2,b-3).

### Correlation between plasma cytokines

A Spearman rank test of correlation among plasma cytokines was performed. There was a significant positive correlation between the following pairs of cytokines in DR-TB: IL-13 and GM-CSF (p = 0.0044, r = 0.4), IL-6 and IL-5 (p = 0.0159, r = 0.4); DS-TB: IL-13 and IL-2 (p = 0.0484, r = 0.3), IL-6 and IL-1β (p = 0.0157, r = 0.4); LTB: IL-12p70 and IL-1α (p = 0.0091, r = 0.4), IL-12p70 and IL-4 (p = 0.0092, r = 0.4), GM-CSF and IL-17 (p = 0.0329, r = 0.3), IL-1α and IL-4 (p = 0.0012, r = 0.5) and IL-13 and IL-5 (p = 0.0183, r = 0.4); HC: IL-12p70 and IL-18 (p = 0.0032, r = 0.5), IL-10 and GM-CSF (p = 0.0457, r = 0.3), GM-CSF and IL-5 (p = 0.0138, r = 0.4), IL-5 and IL-1α (p = 0.0397, r = 0.3), IL-1α and IL-18 (p = 0.0025, r = 0.5), IL-1α and IL-1β (p = 0.0259, r = 0.4), IL-13 and IL-4 (p = 0.0499, r = 0.3), IL-1β and IL-4 (p = 0.0420, r = 0.3), IL-10 and IL-13 (p = 0.0404, r = 0.3), IL-10 and IL-13 (p = 0.0404, r = 0.3), and IL-1α and IL-17 (p =  < 0.001, r = 0.73). Whereas significant negative correlation between the following pairs of cytokines in DS-TB: IL-18 and IL-1α (p = 0.0076, r = − 0.4), IL-13 and IL-5 (p = 0.0369, r = − 0.3), LTB: IL-12p70 and IL-6 (p = 0.0206, r = − 0.4), HC: IL-12p70 and TNF-α (p = 0.0494, r = − 0.3) shown in Fig. 6a–d.

## Discussion

TB biomarkers can be considered crucial to achieve the global TB elimination targets. The effort to understand causal factors like cytokines and their differential expression during different stages of TB is valuable in identifying unique biological signatures^22^. The phenomena of host bodily functions are activated, shaped, and responded through multifunctional cytokines. However, adequate balance of cytokines is cardinal for the appropriate protective responses^20^ as the hypo or hyper responses is often dangerous and progress the disease severity. The essence of balanced production of pro- (IL-17) and anti-inflammatory cytokines (IL-10) for MTB clearance was apparent from the experiment with macaques^17^. The diagnostic utility of the cytokines is proven with QFT-IGRA for MTB infection, but less sensitive to discriminate LTB from active TB^22–24^. Therefore, approaches with cytokine blend grab attention to improve their diagnostic abilities.

Considering this, we attempted to estimate an array of cytokines (14 plex) during latency, drug-sensitive and drug-resistant conditions compared to normal healthy condition. The inclusion of all the stages of TB disease spectrum aids an advantage over previous studies that either lack LTB^25,26^ or DR-TB^8,22^ in revealing the serial increase of cytokines from LTB to DS-TB and DR-TB. This trend marks the hyper immune responses and severity of DR-TB by the accumulation of multiple cytokines (IL-12p70, TNF-α, INF-γ, IL-2, IL-17, IL-6, and IL-10) at higher concentration. Furthermore, the numbers and the levels were tapered in DS-TB (INF-γ, IL-2, IL-17, and IL-6) with a further reduction in LTB (INF-γ, IL-2, and IL-17). The possible reason for the higher IFN-γ levels in DR-TB from our study is due to the IFN-γ demand in macrophages to kill the drug resistant mycobacteria. In addition, the elevated IL-10 levels suggest the loss of balance between pro- and anti-inflammatory cytokines that could cause immune suppression. Similar to our findings, DR-TB groups were previously suggested with increased TNF, IFN-γ and IL-10^26,27^ associated with marked necrosis and resistance to drugs. Mensah et al. stated no differences between the DS-TB and DR-TB with smaller sample size^25^. Though the studies for DR-TB is minimal and with varying cytokine levels, the obtained differences from the current study offer a classical view of better separation of DR-TB from DS-TB, LTB, and HC.

All nucleated cells produce cytokines with either antagonistic or synergistic effects^11^ depends on the infection milieu and the host–pathogen interaction. The release of Th1 and Th17 inflammatory cytokines (IL-2, TNF-α, IFN-γ, IL-12 and IL-17) are correlated with host protective responses against TB infection^27,28^ and that was in connection with MTB clearance and sterilization of granulomas in macaques^17^. The so-called protective cytokines switch to pathological responses due to their overproduction and loss of balance in DR-TB and DS-TB from our study as stated earlier by Kumar et al., 2019 where the type 1 and 17 cytokines assist disease pathology in PTB^8^. The inflammation driven by monocytes are sceptical for pathological response in TB as their partial depletion in patients with chagas disease lessened the pro- (IFN-γ, IL-2 and IL-5) and anti-inflammatory (IL-10) cytokine levels with improved antigen presentation ability of B cells^30^. Peripheral accumulation of monocytes, as evidenced through increased MLR could be the major source of the driving systemic inflammation and, elevating the circulatory cytokine levels among the DR-TB, when compared to the effector T cells. The damage caused by DR-TB is quite long term as the heightened levels of TNF, IFN-γ and IL-12 were sustained even after ATT in DR-TB individuals than DS-TB^31^. Cytokines work in a cascade fashion during TB, where IL-12 controls the Th1 cytokine (IL-2/IFN-γ) production^8,13,32^. The IFN-γ in turn activates macrophages^26,33^ that induce TNF-α secretion for infection containment and mycobacterial growth restriction^26,34,35^. TNF-α and IL-1α are the predominant contributors of granuloma formation^22,36^. However, their protective effect was down regulated by the high IL-6/IL-10 levels in association with pSTAT3/SOCS3 expression which ultimately led to immune suppression and impaired T cell function^37^. In addition, type 1 cytokines (IFN-γ and TNF-α) determines the degree of infection by their discrete association with bacterial density^8^ and turns their personality of protection towards disease dissemination.

Cytokines were extensively investigated during TB disease to understand their association with bacterial burden (IL-17A, IFN-γ, TNF-α and IL-6)^8,38^, cavitation (IL-1β)^19^, disease severity and pathogenesis (IL-17A, IL-1β, and IL-6)^8,9^, and time to culture conversion (IL-17A)^8^. The ideal focus on cytokine patterns determines their biomarker abilities for prognosis, TB detection (IL-2, IFN-γ and TNF-α)^39,40^, discrimination of TB from LTB (TNF, IL-12p40 and IL-17)^41^ and treatment outcome (IL-6, IL-1β and IFN-γ /IL-10 ratio)^38,42,43^. However, the differences reported were varied between the studies and was complexed to find the true candidates. Smaller sample size might mask the true reflection of the disease status as that was apparent from our previous experience with cytokines that contrasted the current observation^44^. Dimensionality reduction of the current data enabled top 8 cytokines (IL-17, IL-1α, IL-2, IL-10, IL-5, IFN-γ, TNF-α and IL-6) with better separation of 4 study groups in different clusters with the accuracy of 94.4%. Moreover, further reduction to the topmost classifiers for DR-TB vs DS-TB (IL-17, IL-5 and TNF-α), DS-TB vs LTB (IL-1α, IL-5 and IL-10) and LTB vs HC (IL-17, IL-2 and IFN-γ) may account for 100% accuracy in discriminating between them.

Though systemic responses don’t reflect the accurate in-vivo status of granuloma^17^, the observations from the current study gives an overview of cytokine behaviour across TB disease spectrum. Since cytokines are multifunctional, the combined effect of cytokines and their signalling network at disease setting must be explored further both in-vitro and in-vivo to understand their intrinsic role in TB pathogenesis. Our study has limitation, that the details of culture conversion and the treatment outcome is unknown. However, our study has advantage over sample size, inclusion, and comparison of TB disease spectrum (DR-TB, DS-TB, and LTB) with healthy controls (HC).

In conclusion, IL-17 exhibited stage specific increase with area under curve value above 0.9 that decipher good sensitivity and specificity across the infection spectrum. Our findings could identify stage specific cytokines, particularly upsurge of specific cytokines was found in DR-TB exhibiting hyper immune responses and disease severity. Future validation of these cytokine signatures in larger cohort at multiple sites may uncover their biomarker potency and their role in host immune system towards drug-resistance.

## Data Availability

The data supporting the findings of this article will be made available by the corresponding author, upon request.

## References

[1] World Health Organization. *Global tuberculosis report 2021*. (World Health Organization, 2021).

[2] Ahmad S, Jaber A-A, Mokaddas E (2007). Frequency of embB codon 306 mutations in ethambutol-susceptible and -resistant clinical Mycobacterium tuberculosis isolates in Kuwait. Tuberculosis.

[3] Pulimood AB, Peter S, Rook GWA, Donoghue HD (2008). In situ PCR for Mycobacterium tuberculosis in endoscopic mucosal biopsy specimens of intestinal tuberculosis and Crohn disease. Am. J. Clin. Pathol..

[4] Yu Y (2012). Different patterns of cytokines and chemokines combined with IFN-γ production reflect mycobacterium tuberculosis infection and disease. PLoS ONE.

[5] Mustafa AS, Al-Saidi F, El-Shamy ASM, Al-Attiyah R (2011). Cytokines in response to proteins predicted in genomic regions of difference of Mycobacterium tuberculosis: Cytokines & RD proteins of M. tuberculosis. Microbiol. Immunol..

[6] Jo E-K, Park J-K, Dockrell HM (2003). Dynamics of cytokine generation in patients with active pulmonary tuberculosis. Curr. Opin. Infect. Dis..

[7] Mihret A (2013). Plasma cytokines and chemokines differentiate between active disease and non-active tuberculosis infection. J. Infect..

[8] Kumar NP, Moideen K, Banurekha VV, Nair D, Babu S (2019). Plasma proinflammatory cytokines are markers of disease severity and bacterial burden in pulmonary tuberculosis. Open Forum Infect. Dis..

[9] Rambaran S (2021). Effect of Inflammatory Cytokines/chemokines on pulmonary tuberculosis culture conversion and disease severity in HIV-infected and -uninfected individuals from South Africa. Front. Immunol..

[10] Chowdhury IH (2014). Alteration of serum inflammatory cytokines in active pulmonary tuberculosis following anti-tuberculosis drug therapy. Mol. Immunol..

[11] Domingo-Gonzalez R, Prince O, Cooper A, Khader S (2016). Cytokines and chemokines in Mycobacterium tuberculosis infection. Microbiol. Spectr..

[12] Druszczyńska M, Godkowicz M, Kulesza J, Wawrocki S, Fol M (2022). Cytokine receptors-regulators of antimycobacterial immune response. Int. J. Mol. Sci..

[13] Cooper AM, Khader SA (2008). The role of cytokines in the initiation, expansion, and control of cellular immunity to tuberculosis. Immunol. Rev..

[14] Walker NF, Meintjes G, Wilkinson RJ (2013). HIV-1 and the immune response to TB. Future Virol..

[15] Tania Beatriz Romero-Adrian, J. L.-M. & Ndez, A. V. Role of cytokines and other factors involved in the Mycobacterium tuberculosis infection. *World J. Immunol.* **5**, 16–50 (2015).

[16] Olsen, A. *et al.* Targeting mycobacterium tuberculosis tumor necrosis factor alpha-downregulating genes for the development of antituberculous vaccines. *mBio* **7**, e01023-15 (2016). 10.1128/mBio.01023-15 PMC4895118 27247233

[17] Gideon HP (2015). Variability in tuberculosis granuloma T cell responses exists, but a balance of pro- and anti-inflammatory cytokines is associated with sterilization. PLOS Pathog..

[18] Torrado E, Robinson RT, Cooper AM (2011). Cellular response to mycobacteria: Balancing protection and pathology. Trends Immunol..

[19] Stek C (2018). The immune mechanisms of lung parenchymal damage in tuberculosis and the role of host-directed therapy. Front. Microbiol..

[20] Cicchese JM (2018). Dynamic balance of pro- and anti-inflammatory signals controls disease and limits pathology. Immunol. Rev..

[21] Rivero-Lezcano O (2008). Cytokines as immunomodulators in tuberculosis therapy. Recent Patents Anti-Infect. Drug Disc..

[22] Yao X (2017). Multiplex analysis of plasma cytokines/chemokines showing different immune responses in active TB patients, latent TB infection and healthy participants. Tuberc. Edinb. Scotl..

[23] Richeldi L (2006). An update on the diagnosis of tuberculosis infection. Am. J. Respir. Crit. Care Med..

[24] Parida SK, Kaufmann SHE (2010). The quest for biomarkers in tuberculosis. Drug Discov. Today.

[25] Mensah GI (2021). Identification of serum cytokine biomarkers associated with multidrug resistant tuberculosis (MDR-TB). Immuno.

[26] Basingnaa A, Antwi-Baffour S, Nkansah DO, Afutu E, Owusu E (2018). Plasma levels of cytokines (IL-10, IFN-γ and TNF-α) in multidrug resistant tuberculosis and drug responsive tuberculosis patients in Ghana. Dis. Basel Switz..

[27] Deveci F, Akbulut HH, Turgut T, Muz MH (2005). Changes in serum cytokine levels in active tuberculosis with treatment. Mediat. Inflamm..

[28] Amiano NO (2022). circulating monocyte-like myeloid derived suppressor cells and CD16 positive monocytes correlate with immunological responsiveness of tuberculosis patients. Front. Cell. Infect. Microbiol..

[29] Cooper AM, Mayer-Barber KD, Sher A (2011). Role of innate cytokines in mycobacterial infection. Mucosal Immunol..

[30] Gomes JAS (2014). Inflammatory mediators from monocytes down-regulate cellular proliferation and enhance cytokines production in patients with polar clinical forms of Chagas disease. Hum. Immunol..

[31] Téllez-Navarrete NA (2021). Anti-tuberculosis chemotherapy alters TNFR2 expression on CD4+ lymphocytes in both drug-sensitive and -resistant tuberculosis: However, only drug-resistant tuberculosis maintains a pro-inflammatory profile after a long time. Mol. Med. Camb. Mass.

[32] Cooper AM (2009). Cell-mediated immune responses in tuberculosis. Annu. Rev. Immunol..

[33] Tötemeyer S (2006). IFN-gamma enhances production of nitric oxide from macrophages via a mechanism that depends on nucleotide oligomerization domain-2. J. Immunol. Baltim. Md.

[34] Mattos AMM (2010). Increased IgG1, IFN-gamma, TNF-alpha and IL-6 responses to Mycobacterium tuberculosis antigens in patients with tuberculosis are lower after chemotherapy. Int. Immunol..

[35] Nemeth J (2011). Specific cytokine patterns of pulmonary tuberculosis in Central Africa. Clin. Immunol. Orlando Fla.

[36] Hernandez-Pando R (1997). Analysis of the local kinetics and localization of interleukin-1 alpha, tumour necrosis factor-alpha and transforming growth factor-beta, during the course of experimental pulmonary tuberculosis. Immunology.

[37] Harling K (2019). Constitutive STAT3 phosphorylation and IL-6/IL-10 co-expression are associated with impaired T-cell function in tuberculosis patients. Cell. Mol. Immunol..

[38] Sigal GB (2017). Biomarkers of tuberculosis severity and treatment effect: A directed screen of 70 host markers in a randomized clinical trial. EBioMedicine.

[39] Casey R (2010). Enumeration of functional T-cell subsets by fluorescence-immunospot defines signatures of pathogen burden in tuberculosis. PLoS ONE.

[40] Harari A (2011). Dominant TNF-α+ Mycobacterium tuberculosis-specific CD4+ T cell responses discriminate between latent infection and active disease. Nat. Med..

[41] Sutherland JS, de Jong BC, Jeffries DJ, Adetifa IM, Ota MOC (2010). Production of TNF-alpha, IL-12(p40) and IL-17 can discriminate between active TB disease and latent infection in a West African cohort. PLoS ONE.

[42] Gupte AN (2022). Baseline IL-6 is a biomarker for unfavourable tuberculosis treatment outcomes: A multisite discovery and validation study. Eur. Respir. J..

[43] Sai Priya VH, Latha GS, Hasnain SE, Murthy KJR, Valluri VL (2010). Enhanced T cell responsiveness to Mycobacterium bovis BCG r32-kDa Ag correlates with successful anti-tuberculosis treatment in humans. Cytokine.

[44] Sampath P (2022). Differential frequencies of intermediate monocyte subsets among individuals infected with drug-sensitive or drug-resistant mycobacterium tuberculosis. Front. Immunol..

